# Molecular evidence for new foodways in the early colonial Caribbean: organic residue analysis at Isla de Mona, Puerto Rico

**DOI:** 10.1007/s12520-023-01771-y

**Published:** 2023-05-03

**Authors:** Lisa Briggs, Jago Cooper, Oliver E. Craig, Carl Heron, Alexandre Lucquin, María Mercedes Martínez Milantchi, Alice Samson

**Affiliations:** 1 Cranfield Forensic Institute, Cranfield University, College Rd, Cranfield, Wharley End, Bedford, MK43 0AL UK; 2 Sainsbury Centre, University of East Anglia, Norfolk Rd, Norwich, NR4 7TJ UK; 3 BioArCH, University of York, Environment Building, Wentworth Way, Heslington, YO10 5NG York UK; 4 Scientific Research Department, The British Museum, Great Russell Street, London, WC1B 3DG UK; 5 Department of Africa, Oceania and Americas, The British Museum, Great Russell Street, London, WC1B 3DG UK; 6 University of Leicester, University Road, Leicester, LE1 7RH UK

**Keywords:** Organic residues, Colonial Caribbean, Pottery, Foodways, Diet, Wine

## Abstract

Ceramic objects account for over 90% of the cultural material recovered from archaeological sites in the Caribbean. However, little research has been conducted on molecular evidence for past food production from these same vessels. Forty ceramic sherds from Isla de Mona have been analysed by GC–MS and GC-C-IRMS in order to address questions surrounding foodways in the Greater Antilles prior to and post European arrival. We evaluate evidence for dietary changes to illuminate aspects of cultural exchange between Indigenous populations and the first generations of Spanish colonists. Here, we show that plant residues are found in a variety of pottery forms, with some evidence for non-ruminant and ruminant fats. The dearth of marine biomarkers is curious given the volume of fish bones found in archaeological contexts on Isla de Mona and may offer evidence for spit-roasting, pit-roasting, or the use of a ‘barbacoa’ to cook fish on the island. The ubiquity of plant residues in a variety of pottery forms may relate to the large-scale cultivation and export of cassava (*Manihot esculenta*) from the island. A Spanish olive jar revealed evidence of wine residues, which may constitute the earliest detection of wine residues in pottery found in the Americas.

**Supplementary information:**

The online version contains supplementary material available at 10.1007/s12520-023-01771-y.

## Introduction

This paper addresses the impact on foodways from the first decades of interaction between the Indigenous peoples of the Greater Antilles and European colonists. It is well known that hunger and food shortages contributed to the failure of the colonists’ first town at La Isabela. Following this disaster, arriving Europeans switched to an almost total dependence on Indigenous food preparation and foodstuffs revealing much about coloniser processes of adaptation and extraction (e.g. Deagan and Cruxent 2002; Deagan 1995). Less is understood about how and when diet and food subsistence strategies changed in later decades and in different contexts such as under the *encomienda* system (the Spanish system of forced labour). To better understand the dynamics of early colonial encounter, we explore molecular evidence for dietary changes detectable in ceramic artefacts from Isla de Mona. Ceramics of Indigenous manufacture are analysed alongside European ceramic vessels by gas chromatography-mass spectrometry (GC–MS) and GC-combustion-isotope ratio mass spectrometry (GC-C-IRMS) in order to investigate the local diet and food subsistence strategies on Isla de Mona and how these changed during the early periods of European colonial presence. This preliminary study will establish the feasibility of future, large-scale investigations into the organic residues present in Caribbean ceramic assemblages. Our research builds on the first GC–MS studies of ceramics from nearby Hispaniola conducted by Vanderveen (Vanderveen 2007, 2006) and expands the scope of such research by including GC-C-IRMS data, gradient analysis of the sherds, and an acid butylation method designed to detect wine residues. Our fundamental research questions include whether lipids survive in these vessels, what these lipids can tell us about diet and food subsistence strategies on Isla de Mona, and to what extent lipids discovered in Indigenous vessels differ from those discovered in European ceramics found on the same island.

### Isla de Mona

**Fig. 1:**
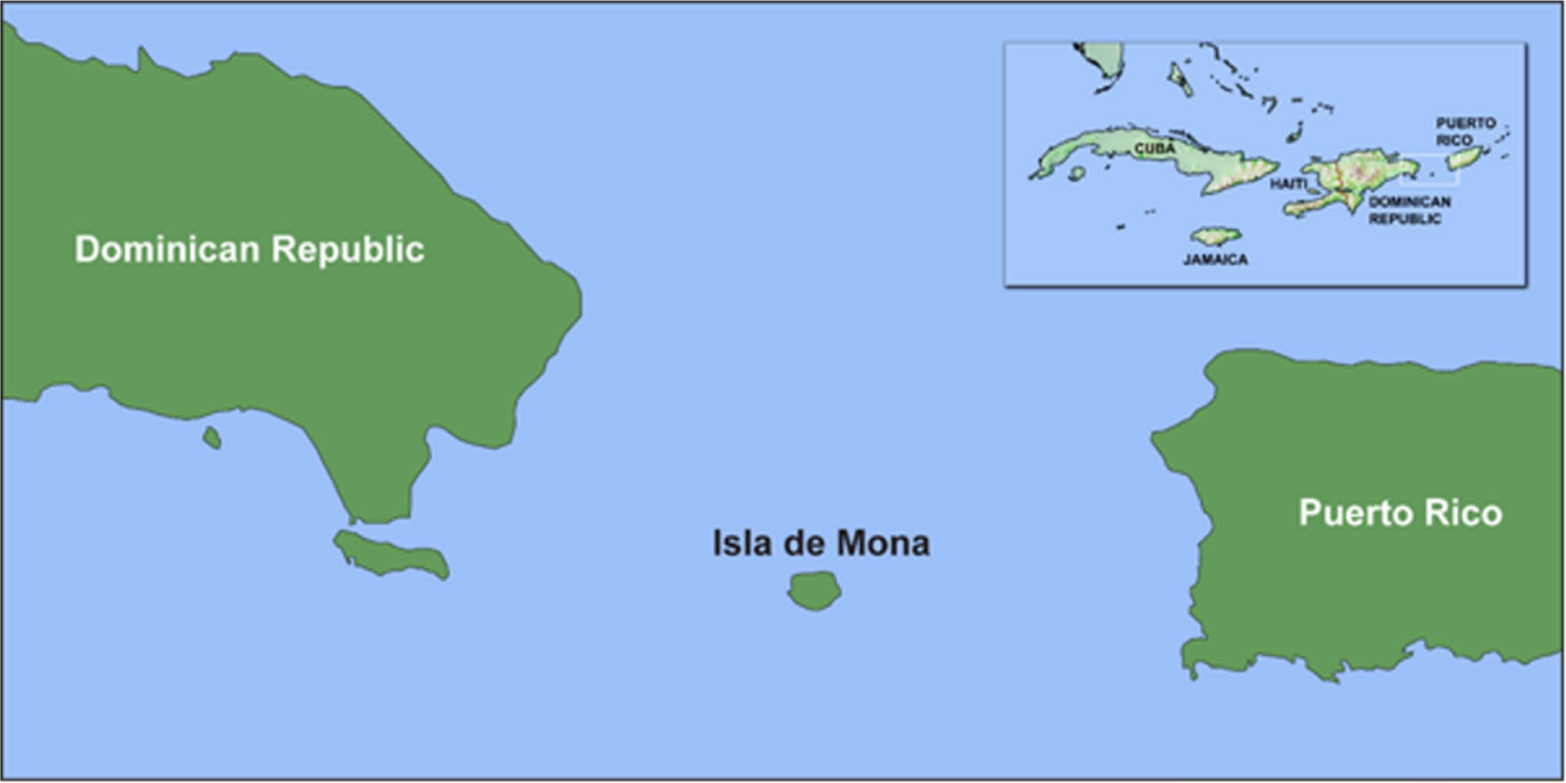
Location of Isla de Mona, situated between modern-day Dominican Republic and Puerto Rico

Isla de Mona is a currently uninhabited island in the northern Caribbean, part of the Puerto Rican archipelago. It is a 50 km^2^ flat-topped carbonate platform with 90-m cliffs sticking out of the fast-flowing sea passage between Puerto Rico and the Dominican Republic, with the island’s centre located at 18.0829° N, 67.8927° W (Fig. 1). Beaches on the south and west coasts provide some anchorage within fringing coral reefs which quickly drop off to depths of up to a thousand metres. Despite being semi-arid at present, with poor soil development, and no permanent sources of freshwater, it is not known whether these adverse conditions were similar in the past. It is known, however, that Isla de Mona attracted people to it for over 5000 years, in keeping with early patterns of small island use by Indigenous people across the archipelago (Keegan et al. 2008). As well as for the island’s abundant seasonal marine and terrestrial resources, people were drawn underground into its extensive cave systems. Archaeological research has documented pre-Columbian and early colonial Indigenous rock art throughout the caves, at least two ballcourts in the island’s interior, and a village site on the west coast (Dávila Dávila 2003; Lace 2013; Rouse 1952; Samson and Cooper 2015). In the sixteenth century, the Spanish Crown established a royal encomienda or provisioning station on Mona, exploiting Indigenous labour to supply food and textiles to its burgeoning mining industries across the region (Samson et al. in press). In later centuries, the island became a base for pirates and refugees and, much later, guano miners in the nineteenth century.

### Evidence for diet and foodways in the pre-Columbian Caribbean

Pottery accounts for around 90% of all pre-Columbian artefacts (Keegan 2000, p. 135); however, the traditional focus in archaeology has been on constructing typo-chronologies rather than what it can tell us about diet. Increasingly, scientific techniques are being applied to elucidate this (Vanderveen 2007, 2006). To date, studies of diet in the region have used stable isotope analysis of human bone collagen (Pestle 2010, 2013; Pestle and Colvard 2012), identification of starch grains through scanning electron microscopy (Ciofalo et al. 2019, 2018; Pagán-Jiménez and Mickleburgh 2022), and zooarchaeology through the identification of faunal remains (Keegan 2000; Rainey 1940). We believe analysis of pottery contents through organic residue analysis can provide information not obtainable by these methods.

Stable isotope analysis of skeletal remains from Puerto Rico has provided insights into shifts in diet in the Indigenous population over time. However, stable isotope analysis of human bone collagen can only illuminate the diet of the specific individuals under study, relies on the successful preservation and extraction of human bone collagen (which can quickly degrade in tropical environments), and cannot distinguish between the consumption of meat and dairy products. Pottery, on the other hand, could provide information about productive and communal activities. GC–MS and GC-C-IRMS analysis of pottery can offer a greater specificity by distinguishing between plant, dairy, marine, and ruminant versus non-ruminant foodstuffs.

Analysis of starch grains by scanning electron microscopy indicates high levels of Indigenous manioc/cassava consumption (Ciofalo et al. 2019, 2018; Pagán-Jiménez and Carlson 2014); however, this analytical method can only detect the remains of food products with a starchy, granular structure. Analysis of the lipids trapped in pottery could help to fill in the gaps left by this type of analysis.

Faunal remains provide important dietary information on archaeological sites. For example, the site of Coralie on the island of Grand Turk yielded abundant faunal remains, and a subsistence shift from the large-scale consumption of local land crabs to marine molluscs has seen more widely across the region. This is concomitant with a change in pottery styles from the Saladoid style (c. 500 BCE to 600 CE) to the Ostionoid style (c. 600–1500 CE) (Keegan 2000, p. 152). Froelich Rainey (1940) perceived this as evidence for two distinct cultures: a ‘Crab Culture’ and a ‘Shell Culture’, yet later research has shown these differences are paralleled in other areas of material culture (Rodríguez-Ramos 2005). This offers insight into the dynamic nature of food pathways in the Caribbean, providing a clear illustration that the diet of Indigenous populations did not remain static, but rather evolved over time. Given the variety of diets prior to contact with Spanish colonists, our research seeks to understand what further changes may have occurred in diet and culinary practices after early interactions with Europeans.

In neighbouring regions, organic residue analysis by GC–MS has produced significant findings: residue studies of Native American ceramics from the Midwest, Mississippi River, and the American Bottom regions of continental North America have revealed molecular evidence for varying levels of maize consumption (Reber and Evershed 2004), thus contributing to our understanding of agriculture, dietary practices, and culinary traditions. Two publications characterise the lipids recovered from Indigenous vessels from Hispaniola by GC–MS (Vanderveen 2007, 2006), and none have been conducted on ceramics from Puerto Rico. Vanderveen reported evidence for a variety of foodstuffs in the 55 sherds recovered from seven archaeological sites on Hispaniola, including ruminants, conifer products, and plant products (2006, pp. 121–156), yet without additional analysis by GC-C-IRMS, it is difficult to pinpoint a precise source for the lipids detected. While the Vanderveen studies provided an important first step in the molecular analysis of Indigenous pottery from the Greater Antilles, more work is needed in this area.

At the pre-Columbian and colonial era site of Sardinera on Isla de Mona, there is faunal evidence that can help us infer aspects of diet. Our excavations of the sixteenth century deposits recovered fish and marine molluscs as well as pig bones (*Sus scrofa domesticus*). In earlier excavations, Rouse (1952, p. 368) makes brief mention of native bird, crab, fish, hutía, iguana, manatee, turtle, and molluscs amongst the excavated food remains. He also tentatively identified cow and another imported mammal in upper layers. Food remains were the most abundant category in Dávila Dávila’s excavations at the same site (2003, p. 139); the majority of which (60%) were marine molluscs in addition to fish and hutía, with cow, goat, and pig in the top layer (0–20 cm) (2003, pp. 145–154). The floor of the nearby site of Cueva Campanita, a collapsed dolina on the cliffs above Sardinera, was covered in remains of marine molluscs and large mammal bones during our visit in 2016, and Dávila Dávila reported marine molluscs and remains of cow, goat, and pig on the surface during earlier surveys (2003, pp. 166–167). Although these remains could be associated with the long use of this place into more recent times, Indigenous and sixteenth-century Spanish pottery is also abundant. In terms of the historic evidence, Spanish accounts repeatedly emphasise the fertility and food abundance on the island including cassava, beans, maize, peppers, fruit, and fish, turtles, crabs, and the best-tasting iguana in the Americas (*Cyclura cornuta stejnegeri*) (Fernandez de Oviedo y Valdés, 1851, bk. XII, chap. VII, p.394; book XVI, chap. I, p. 465; Las Casas 1875, p. XCVIII, 68–69; Wadsworth 1973). Isla de Mona became a centre for food production and a major exporter of cassava bread in the sixteenth century as one of three Spanish Crown-controlled supply stations in Puerto Rico producing food and bedding (hammocks) for pearl and gold-mining industries (Samson et al. in press).

## Materials and methods

### Ceramic artefacts

Ceramic artefacts from two archaeological sites on the island, Playa Sardinera and Cueva Campanita, are analysed here to better understand what changes may have occurred in dietary practices and food preparation at colonial sites. Archaeological evidence suggests that occupation at these sites ceased after 1560 CE. Forty ceramic artefacts were selected for analysis by GC–MS, consisting of 14 sherds of Iberian origin, and 26 sherds of Indigenous origin (Table 1).

**Table 1:** Ceramic types analysed, time periods of deposition, number of samples in each group, and percentage of the total (*n* = 40)

Category	Time Period	Total pot sherds
Chican-Ostionoid	1300–1560	9
Indigenous undecorated	1300–1560	14
Buren	1300–1560	3
European glazed	1494–1560	10
Olive jar	1494–1560	2
Green bacin	1494–1560	2

**Fig. 2:**
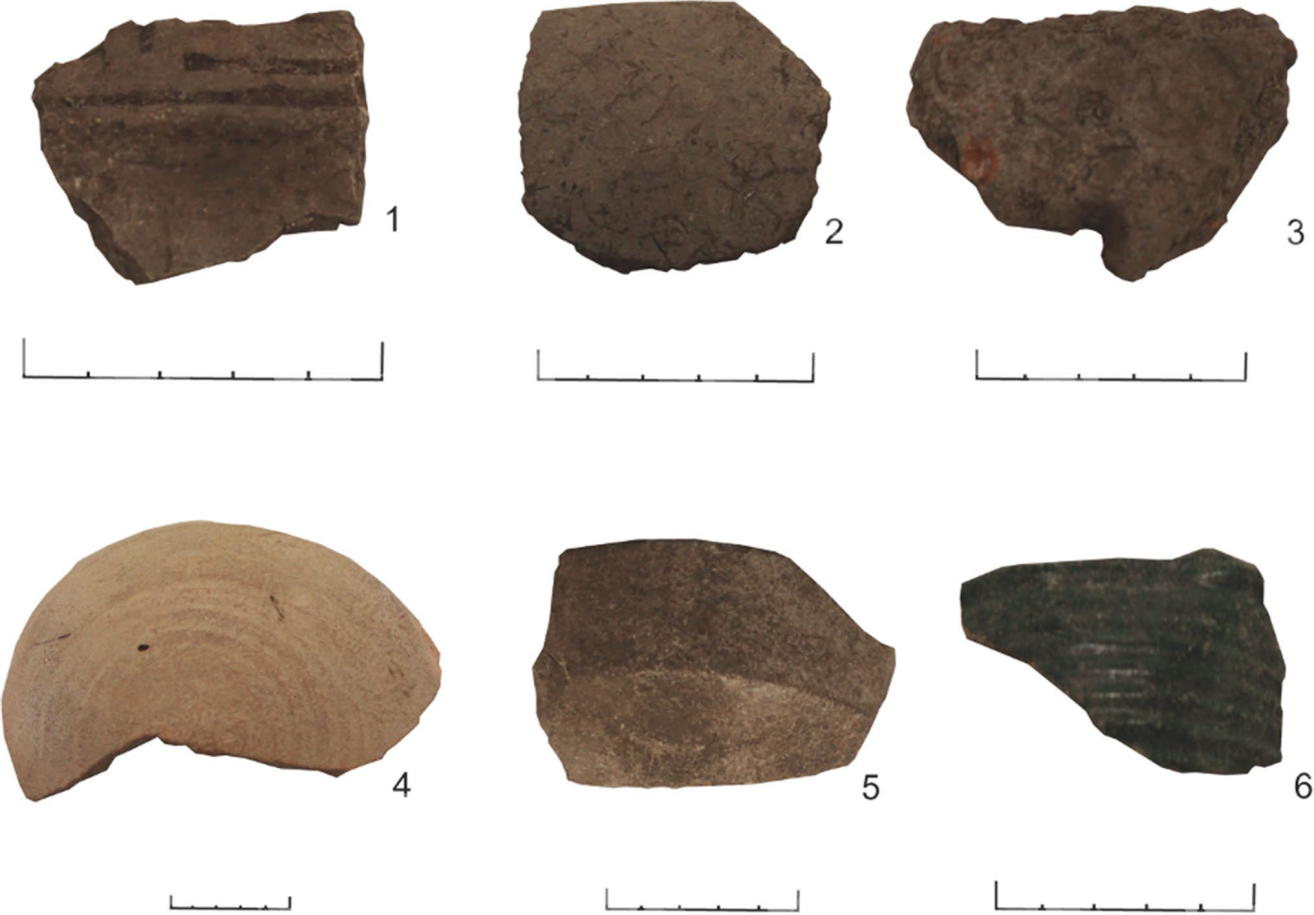
Ceramic samples from the six categories of pottery analysed. 1 = Chican-Ostionoid (217), 2 = Indigenous undecorated (231), 3 = buren (220), 4 = Spanish olive jar (175), 5 = European glazed (227), 6 = green bacin (229). Scale = 5 cm

There are six broad ceramic types represented in this sample set (Table 1, Fig. 2). The 26 Indigenous sherds fall into three groups: undecorated plain bowls and plates, decorated bowls and plates of the Chican-Ostionoid series, and buren, which are flat, ceramic griddles used for cooking a variety of plants and other foods (Rouse 1992, p. 12). Two of the European sherds derive from ‘olive jars’, a large ceramic amphora used to transport foodstuffs to the Americas on board ships. While the term olive jar implies that these vessels contained either olives or olive oil, olive jars are likely to have been multiuse vessels which contained a range of other liquid commodities such as wine (Goggin 1960). This supposition is investigated here. Other vessels of European manufacture found at Isla de Mona and analysed here consist of thick-walled open forms with an emerald-green glaze on both the interior and exterior surface and have been identified as green bacin/green lebrillo, which are large (50-cm diameter), rimmed platters. A number of imported sherds are covered in a white or brown glaze. Of the 10 glazed sherds of European manufacture, seven have a whitish/tan coloured glaze and may belong to the ‘Columbia plain’ ceramic type, but this assignation is tentative due to the small size of the sherds and poor preservation of the glaze. The remaining three are of an unidentified European type with a slightly browner shade of glaze than the other seven. As the condition of the glazed ceramics is not sufficient for a secure typological identification, these have been grouped together as ‘European glazed’ and are listed as either European white-glaze or European brown-glaze throughout. The original shape of the glazed vessels is not clear, but they were most likely plates or shallow bowls (escudillas) given the distance between the rim and the base (i.e. Fig. 2, sherd 5). The assemblage represents a mixture of individual and communal food storage (olive jar), food preparation (bacin, buren, bowls), and food consumption (plates, bowls) forms. Although there is no historical record for European presence on the island until 1508, the date ranges for European material in Table 1 reflect the possibility of indirect acquisition through Indigenous networks.

The relatively small number of samples (26 Indigenous and 14 European) allows us to draw preliminary interpretations from our GC–MS investigations which can be supplemented in the future in a larger study that incorporates a greater number of ceramic samples.

### Analytical methods

#### GC–MS methods

In an effort to detect a wide range of compounds, all sherds were extracted at least twice with two different methods of extraction. This included the acidified methanol extraction method which utilises methanol and sulphuric acid to aggressively target lipids in low concentrations and by solvent extraction using dichloromethane and methanol (2:1) designed to preserve larger molecules such as triacylglycerols. These were prepared and performed according to established protocols (Correa-Ascencio and Evershed 2014; Drieu et al. 2021; Dudd and Evershed 1998; Stern et al. 2000). A test was conducted on a subset of four sherds in order to assess levels of environmental input into the ceramics. This consisted of obtaining and separately extracted five successive layers from the interior of the four vessels to evaluate qualitative/quantitative differences in the lipid extracted according to a previously published protocol (Stern et al. 2000). In order to assess the presence of wine biomarkers, we investigated a subset of seven samples with a third extraction method using an acid butylation protocol designed to detect tartaric acid and malic acid (Drieu et al. 2021; Garnier and Valamoti 2016). Detailed information on the preparation of all samples for GCMS analysis is provided in the Supplementary Information.

#### GC-C-IRMS methods

In addition to GC–MS analysis, 25 samples have been analysed by GC-C-IRMS. This technique, first introduced in the 1990s, has made it possible to access stable isotope information from individual biomarker molecules, making further information available in the application of organic residue analysis in archaeology, including the differentiation between adipose and dairy fats (Evershed et al. 1997, 1994). Based on the concentration of C_16:0_ (palmitic acid) and C_18:0_ (stearic acid) in the extracts, a subset of 25 ceramics were selected for analysis by GC-C-IRMS. In order to assess the presence or absence of biomarkers associated with marine resources, the 25 samples selected for GC-C-IRMS were also analysed by GC–MS in selected ion monitoring (SIM) mode and using a temperature programme setup to better detect and resolve the isoprenoid (i.e. 4,8,12-TMTD, pristanic and two natural diastereomers of phytanic acids) and the *ω*-(o-alkylphenyl)alkanoic acids (thereafter referred to as ‘Aqua-SIM’)(Shoda et al. 2017). Detailed information on the preparation of all samples is provided in the Supplementary Information.

## Results

Lipid recovery rates were high overall, with all sherds analysed in this study yielding above the minimum 5 ug/g threshold for interpretable lipid profiles (Evershed 2008; Heron et al. 1991). Indigenous undecorated vessel 183 had the lowest concentration of lipids at 14 µg/g, and European white glazed vessel had the highest at 259 µg/g; however, both Indigenous and European vessels are amongst the top three highest and lowest lipid concentrations. No clear pattern has emerged in terms of lipid concentration and vessel type.

There is little evidence for contamination in these samples, with two exceptions. The first is the presence of diethyltoluamide detected in sample 216. This compound, also known as DEET, is the active ingredient in many insect repellents and likely derives from post-excavation handling. The second possible indication of contamination is the presence of even-numbered *n-*alkanes. The presence of even numbered *n-*alkanes dominating over odd has been interpreted as contamination from petroleum products (Freeman and Pancost 2014; Whelton et al. 2018) or possibly the result of thermal alteration of organic matter present in the deposition environment (Bondetti et al. 2020; March et al. 2014; Suryanarayan et al. 2021; Wang et al. 2017).

**Fig. 3:**
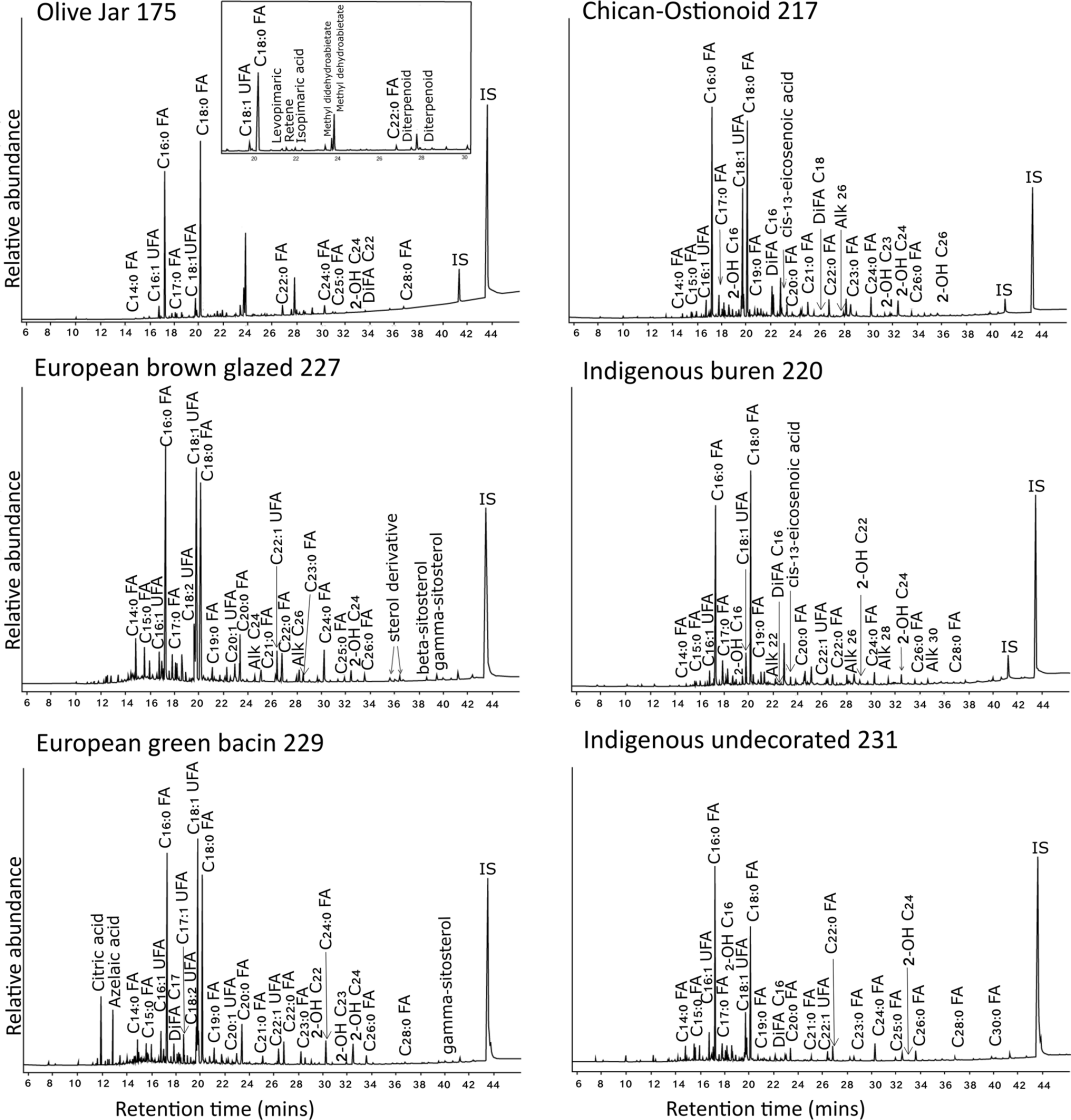
Characteristic chromatograms for the six ceramic types analysed in this study consisting of Spanish olive jars, Chican-Ostionoid, Indigenous undecorated, green bacin, European glazed, and buren

A wide variety of compounds were detected in the samples analysed. The most abundant are a broad range of free fatty acids, including both saturated (FA) and unsaturated (UFA), and of a wide range of carbon lengths from short to long-chain fatty acids (LCFA) and very-long-chain fatty acids (VLCFA). Additional compounds detected include dicarboxylic acids (diacids), hydroxy fatty acids, phytosterols, fatty alcohols, and *n-*alkanes. Characteristic chromatograms for the six ceramic types analysed here are shown in Fig. 3.

### Lipid profiles

**Fig. 4:**
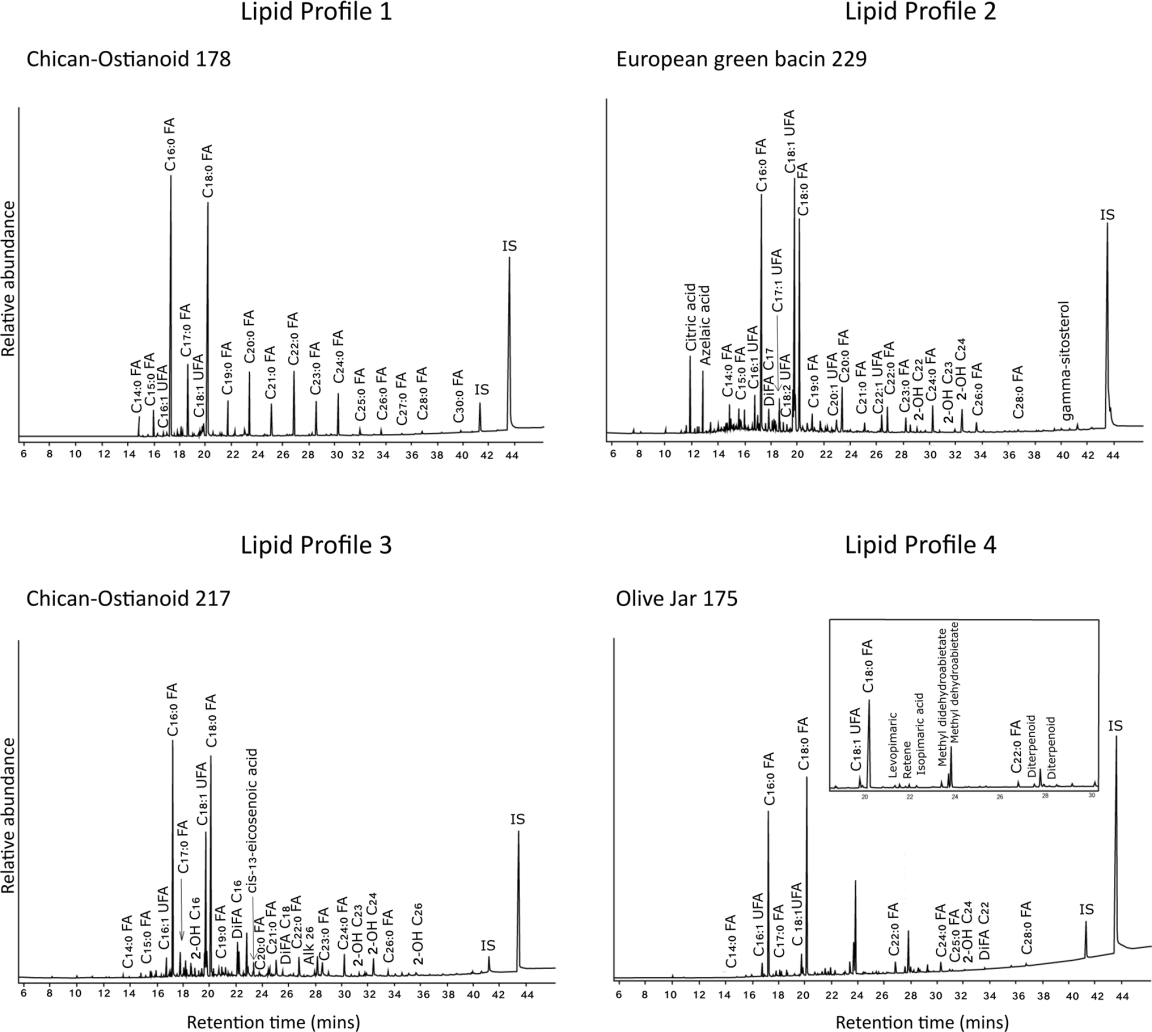
Examples of Lipid Profiles 1–4 shown by chromatograms characteristic of each group

Lipid biomarker analysis by GC–MS has shown the residues from these vessels fall into at least four, and possibly more, broad categories (Table 2). Identifying ‘lipid profiles’ allows us to demonstrate overarching patterns amongst ceramic samples, which demonstrate similar molecular results (e.g. Dunne et al. 2022a, b). An example of each lipid profile is shown in Fig. 4. A table detailing the main results found for each individual sample, their pottery type, lipid profile, lipid concentrations, and ẟ^13^C values found in the GC-C-IRMS analysis are presented in Table 3.

**Table 2:** Overview of lipid profiles detected by acidified methanol extraction, with main molecules and compound classes detected, origin of ceramics, ceramic types in each lipid profile, and number of samples assigned to each group

Lipid profile	Main molecules	Origins	Vessel types in this profile	Interpretation	Number of samples assigned
1	C_18_, C_16_, LCFA (C_14_–C_20_), VLCFA (C_21_–C_32_)	Indigenous and European	Chican-Ostionoid, green bacin, white glazed, undecorated, buren	Plant and animal products	*n* = 12
2	C_16_, C_18_, High UFAs (C_18:1_, C_16:1_, C_22:1_)	Indigenous and European	Chican-Ostionoid, green bacin, white glazed, brown glazed, undecorated, buren	Plant (possible *Brassica*) and animal products	*n* = 10
3	C_16_, C_18_,odd-numbered alkanes, diacids, hydroxy fatty acids	Indigenous and European	Chican-Ostionoid, green bacin, brown glazed, undecorated, buren	Plant products	*n* = 16
4	C_16_, C_18_, diterpenoids	European	Olive jars	Conifer products and wine residues	*n* = 2

**Table 3:** Sample number, origin of sample (Indigenous of European), site on Isle de Mona, ceramic type, lipid concentration, ẟ^13^C and Δ^13^C, lipid profile, assignation, and the presence or absence of LCFA and/or diacids.

Sample number	Origin	Site	Ceramic type	Lipid concentration ug/g	δ^13C^ C_16:0_	δ^13C^ C_18:0_	Δ^13C^(C_18:0_-C_16:0_)	Lipid profile	Assignation	LCFA (x) and/ or diacids present (o)
175	European	Cueva Campanita	Olive jar	27.73	-27.71	− 28.30	− 0.59	4	Wine and conifer products	x
176	European	Cueva Campanita	Green bacin	15.27	-	-	-	1	Plant + degraded animal fats	x
177	Indigenous	Cueva Campanita	Undecorated	136.15	− 21.82	− 22.39	− 0.58	3	Plant	x
178	Indigenous	Playa Sardinera	Chican-Ostionoid	44.63	− 19.40	− 20.20	− 0.80	1	Plant + degraded animal fats	x
179	Indigenous	Playa Sardinera	Undecorated	53.77	-	-	-	3	Plant	x
180	Indigenous	Playa Sardinera	Chican-Ostionoid	22.70	-	-	-	1	Plant + degraded animal fats	x
181	Indigenous	Playa Sardinera	Undecorated	29.53	-	-	-	3	Plant	x
182	Indigenous	Playa Sardinera	Chican-Ostionoid	14.27	-	-	-	3	Plant	x
183	Indigenous	Playa Sardinera	Undecorated	14.13	-	-	-	3	Plant	xo
184	Indigenous	Playa Sardinera	Undecorated	28.97	− 23.70	− 23.26	0.44	1	Plant + degraded animal fats	x
185	European	Playa Sardinera	Olive jar	30.94	− 27.09	− 28.43	− 1.34	4	Wine and conifer products	x
200	Indigenous	Playa Sardinera	Chican-Ostionoid	214.35	− 24.52	− 24.77	− 0.26	1	Plant + degraded animal fats	x
201	European	Playa Sardinera	Green basin	73.68	-	-	-	3	Plant	x
209	European	Playa Sardinera	White glazed	27.90	-	-	-	1	Plant + degraded animal fats	x
210	Indigenous	Playa Sardinera	Chican-Ostionoid	56.78	-	-	-	1	Plant + degraded animal fats	x
211	European	Playa Sardinera	White glazed	134.63	− 29.41	− 29.80	− 0.38	2	Plant (possible *Brassica*)	xo
212	European	Playa Sardinera	White glazed	67.33	-	-	-	1	Plant + degraded animal fats	x
213	Indigenous	Playa Sardinera	Chican-Ostionoid	36.54	− 29.29	− 29.56	− 0.27	3	Plant	xo
214	Indigenous	Playa Sardinera	Undecorated	42.40	− 29.29	− 29.14	0.15	1	Plant + degraded animal fats	x
215	Indigenous	Playa Sardinera	Chican-Ostionoid	46.05	− 28.87	− 29.35	− 0.49	2	Plant (possible *Brassica*)	xo
216	European	Playa Sardinera	White glazed	37.27	− 28.04	− 28.36	− 0.32	2	Plant (possible *Brassica*)	x
217	Indigenous	Playa Sardinera	Chican-Ostionoid	64.84	− 27.60	− 28.64	− 1.04	3	Plant	xo
218	European	Playa Sardinera	White glazed	259.27	− 27.05	− 28.56	− 1.51	2	Plant (possible *Brassica*)	xo
219	European	Playa Sardinera	Green basin	63.07	− 28.59	− 29.10	− 0.51	3	Plant	x
220	Indigenous	Playa Sardinera	Buren	45.18	− 29.06	− 29.76	− 0.71	2	Plant (possible *Brassica*)	xo
221	Indigenous	Playa Sardinera	Chican-Ostionoid	30.22	-	-	-	3	Plant	xo
222	Indigenous	Playa Sardinera	Chican-Ostionoid	34.43	-	-	-	3	Plant	xo
223	European	Playa Sardinera	White glazed	31.87	− 27.11	− 26.85	0.27	2	Plant (possible *Brassica*)	x
224	Indigenous	Playa Sardinera	Buren	24.51	-	-	-	3	Plant	xo
225	European	Playa Sardinera	White glazed	43.29	-	-	-	1	Plant + degraded animal fats	x
226	European	Playa Sardinera	Brown glazed	29.81	− 24.94	− 25.97	− 1.04	3	Plant	xo
227	European	Playa Sardinera	White glazed	50.80	− 26.48	− 27.35	− 0.87	2	Plant (possible *Brassica*)	x
228	Indigenous	Playa Sardinera	Undecorated	21.95	-	-	-	3	Plant	xo
229	European	Playa Sardinera	Green basin	34.98	− 27.07	− 28.06	− 0.99	2	Plant (possible *Brassica*)	x
230	Indigenous	Playa Sardinera	Buren	24.94	-	-	-	1	Plant + degraded animal fats	x
231	Indigenous	Playa Sardinera	Undecorated	22.53	− 26.60	− 28.18	− 1.58	2	Plant (possible *Brassica*)	x
232	Indigenous	Playa Sardinera	Chican-Ostionoid	71.51	− 29.81	− 30.04	− 0.23	3	Plant	xo
233	Indigenous	Playa Sardinera	Undecorated	44.08	− 25.97	− 27.83	− 1.86	2	Plant (possible *Brassica*)	xo
234	Indigenous	Playa Sardinera	Undecorated	35.30	-	-	-	1	Plant + degraded animal fats	x
235	Indigenous	Playa Sardinera	Undecorated	32.69	− 29.47	− 30.17	− 0.69	3	Plant	x

The lipid profiles have been assigned in order of specificity (Table 2). Broadly, Lipid Profile 1 could be considered the most generic as the compounds found in this group are present in a wide variety of foodstuffs. Lipid Profile 2 features unsaturated fatty acids in high abundance, including erucic acid (C_22:1_). Lipid Profile 3 contains long-chain dicarboxylic fatty acids, hydroxy fatty acids, and odd-numbered *n-*alkanes. Lipid Profile 4 contains a suite of diterpenoids, with a clear source attributable to conifer products from the *Pinaceae* family.

‘Lipid Profile 1’ is dominated by common fatty acids found in virtually all foodstuffs: palmitic acid (C_16:0_) and stearic acid (C_18:0_), with additional peaks assigned to a series of long-chain fatty acids (LCFA) between 14 and 20 carbon atoms, and very long-chain fatty acids (VLCFA) between 21 and 32 carbon atoms. Twelve out of the 40 sherds fall into this category which accounts for 30% of the samples analysed and includes both Indigenous and European sherds (Table 3). While this profile could be indicative of degraded animal fats (Dudd et al. 1998; Whelton et al. 2018), experimental analysis on modern animals fats and plant oils has shown that with such ubiquitous compounds, it is difficult to distinguish between lipids derived from animals and those derived from plants (Steele et al. 2010). The VLCFA observed in these samples is likely derived from plant waxes.

‘Lipid Profile 2’ contains multiple examples of unsaturated fatty acids (UFAs) in high levels: primarily oleic acid (C_18:1_), palmitoleic acid (C_16:1_) and erucic acid (C_22:1_), with some samples in this profile also containing margaroleic acid (C_17:1_), linoleic acid (C_18:2_), and gadoleic acid (C_20:1_). Oleic acid is also present in high abundance in these samples. Oleic acid accounts for between 56 and 86% of fresh olive oil (Colombini and Modugno 2009, p. 7); however, this UFA is also present in other fats and oils and is a constituent of modern hand creams and cosmetics (Whelton et al. 2021) and could be a sign of contamination. Some samples in this category contain even-numbered *n-*alkanes (samples 216, 218, and 220), which may be indicative of contamination from petroleum products. Two of the samples in this profile also contain phytosterols: European white-glazed 227 contains campesterol and sitosterol, while European green bacin 229 contains sitosterol. Both of these phytosterols have an unambiguous origin in plants (Colombini and Modugno 2009, p. 54). Vegetable oils contain a greater amount of UFAs than animals fats and are therefore more prone to oxidation and polymerisation (Colombini and Modugno 2009, p. 100). As such, these results are somewhat unexpected given the propensity for unsaturated fatty acids to degrade in archaeological ceramics. The preservation of higher concentration of UFAs in these samples may be due to the relatively recent age of the samples (when compared to ceramics commonly investigated by organic residue analysis) or a very high level of UFAs present in the pottery prior to deposition. A third possibility is that these samples contained food products with high levels of antioxidants which may have contributed to the preservation of UFAs. Erucic acid (C_22:1_) is present in all samples of this group and has been identified as an indicator of plants from the *Brassica* family (Colombini and Modugno 2009, p. 7) although this compound is also found in a much wider range of plants. Nine of the 10 samples assigned to this group contain additional compounds indicative of plants, including diacids and hydroxy fatty acids.

‘Lipid Profile 3’ comprises samples with either odd-numbered *n-*alkanes, long-chain dicarboxylic acids, 2-hydroxy fatty acids, or in many cases a combination of these compounds. These three classes of compounds are diagnostic for plant-derived foodstuffs. Plant sphingolipids, for example, are mostly composed of 2-hydroxy fatty acids (Ukawa et al. 2022), and these compounds are widely present in the samples in Lipid Profile 3. Plant waxes such as Candelilla wax are produced by leaves of various *Euphorbia* species which are native to the Americas and are present on Isla de Mona today and contain a mixture of odd-numbered *n-*alkanes with 29–31 carbon atoms (Colombini and Modugno 2009, p. 99). Four samples in this category display such odd-numbered *n-*alkanes, all of Indigenous origin. The two classes of fatty acids, dicarboxylic acids (also known as diacids), and hydroxy fatty acids are common degradation products of other foodstuffs, particularly plant-derived lipids. Diacids can be formed through the uptake of oxygen at the double bond sites on the carbon chain of unsaturated fatty acids (Colombini and Modugno 2009, p. 308). Mono- and polyunsaturated fatty acids (present in plant oils and marine oils) are particularly labile and prone to oxidation and thermal alterations that can produce hydroxy, dihydroxy, dicarboxylic fatty acids, and *⍵*-(o-alkylphenyl)alkanoic acids (APAAs) (Bondetti et al. 2020; Cramp and Evershed 2013; Whelton et al. 2021). There is some overlap between ‘Lipid Profile 2’ and ‘Lipid Profile 3’ as 9 out of 10 samples in Lipid Profile 2 contain both high levels of unsaturated fatty acids and dicarboxylic acids and hydroxy fatty acids. Lipid Profile 3 lacks the high levels of UFAs found in Lipid Profile 2; however, this may be due to partial oxidation or thermal degradation of the unsaturated fatty acids in question.

‘Lipid Profile 4’ comprises sherds with a suite of diterpenoids detected, in addition to the palmitic acid (C_16:0_), and stearic acid (C_18:0_) present in every sample. Diterpenoids are resinous compounds primarily found in conifers of the *Pinaceae* family (Heron and Pollard 1988; Pollard and Heron 1996). The terpenoids detected consist of unaltered diterpenoids including levopimaric acid and isopimaric acid, as well as diterpenoids that have been oxidised or degraded including dehydroabietic acid, 7-oxo-dehydroabietic acid, and finally the molecule retene which is a heating marker which can be indicative of pitch and tar production through the distillation of resinous woods (Connan and Nissenbaum 2003; Heron and Pollard 1988; Pollard and Heron 1996). The two samples in this category are the only two unglazed ‘olive jars’ included in this data set, indicating that both vessels had a resinous lining derived from conifer products.

### Acid butylation

**Fig. 5:**
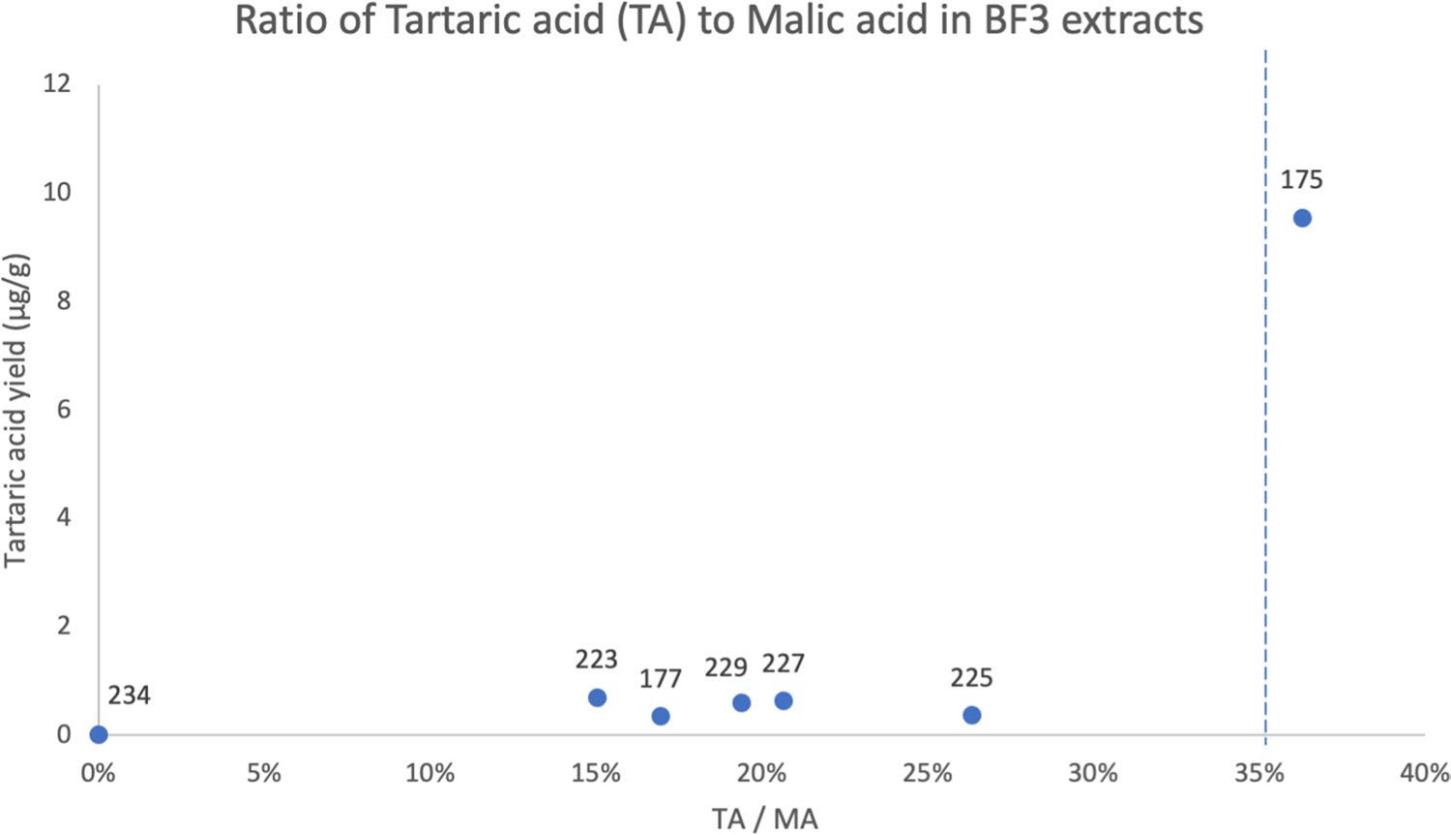
Ratio of tartaric acid to malic acid found for seven samples selected for acid butylation. Blue dotted line at 35% is the minimum threshold for wine products (Drieu et al., 2020)

A subset of seven samples was selected for extraction by acid butylation/boron trifluoride. This method is designed to detect fruit acids including tartaric acid and malic acid, two acids found in grape-based products such as wine (Garnier and Valamoti 2016). Recent analysis of control samples has shown that while a variety of fruits contain tartaric acid and malic acid, it is only in ripened grape products, including wine, and tamarind that a minimum threshold of 35% tartaric acid to malic acid is expressed (Drieu et al. 2021). We calculated the ratio of tartaric acid to malic acid for the seven samples extracted by this method, and results are presented in Fig. 5.

The results show that sample 175, a Spanish olive jar, contains levels of tartaric acid that indicate a past content of wine. This result is consistent with the suite of diterpenoids also found in this samples, as many wine vessels were lined with conifer products in the past in order to more effectively seal the permeable sides of the vessel and contribute antimicrobial properties to the wine (Briggs et al. 2022; Drieu et al. 2021; Heron and Pollard 1988; Pecci et al. 2017, 2013; Pollard and Heron 1996). Tamarind products can also impart a similar ratio of tartaric acid to malic acid in ceramic vessels (Drieu et al. 2021, p. 5); however, tamarind was not present in this region during this time period, having been transported to Mexico and Central America for the first time later in the sixteenth century by Spanish and Portuguese colonists from tropical regions of Africa (Tamale et al. 1995).

Five additional samples (European white glazed 225, European white glazed 227, European green bacin 229, Indigenous undecorated 177, European white glazed 223) yielded low levels of tartaric acid and a lower ratio of tartaric acid to malic acid, possibly indicating a past content of another type of fruit. No tartaric acid was detected in the Indigenous undecorated vessel 234.

### Detection of biomarkers associated with aquatic products

Further GC–MS analysis was undertaken on 25 of the 40 sherds included in this study in order to assess the presence or absence of biomarkers indicative of marine or freshwater fish resources. These biomarkers include isoprenoid fatty acids and *⍵*-(o-alkylphenyl)alkanoic acids (APAAs) with a carbon chain > 20, which are formed through the heating of the polyunsaturated fatty acids (PUFAs) found in marine resources (Bondetti et al. 2020; Cramp and Evershed 2014). APAAs with a carbon chain length of < 20 are created through the heating of foods that contain UFAs, such as vegetable oils, and terrestrial adipose fats (Bondetti et al. 2021; Evershed 2008; Heron and Evershed 1993). In order to further assess the presence of isoprenoid alkanoic acids, the ratio of two naturally occurring diastereomers of phytanic acid, 3S,7R,11R,15-phytanic acid (SRR) and 3R,7R,11R,15-phytanic acid (RRR), were calculated, with a ratio of SRR% over 75.5% indicative of aquatic oil (Lucquin et al. 2016; Schröder and Vetter 2010; Shoda et al. 2017). While phytanic acid is found in both aquatic and ruminant animals, a higher SRR/RRR ratio (SRR%) is characteristic of lipids derived from aquatic organisms (Shoda et al. 2017, p. 169).

Six of the 25 samples analysed revealed an SRR% above the 75.5% threshold for aquatic oils: five are of Indigenous origin (samples 178, 184, 213, 229, and 233), and the sixth is a sherd of European manufacture thought to belong to the Colombian plain type (sample 216). The sherds of Indigenous origin include both decorated and undecorated vessels. Eleven of the 25 samples revealed traces of *⍵*-(o-alkylphenyl)octadecanoic acid (APAA C_18_), yet none revealed APAAs with a carbon chain length of < 20. We also determined whether or not APAAs with a carbon chain length of < 20 would be detectible by these methods by establishing the limit of detectability and found that if present, these larger chain length APAAs should be detected, meaning that the APAA C_18_ detected was not based on random results or machine noise. APAA C_18_ is readily created from the heating of a wide variety of foodstuffs including vegetable oils and terrestrial adipose tissues; this identification is of limited diagnostic value in archaeological material (Bondetti et al. 2020, p. 595). Further information can be gathered by the distribution of the APAA C_18_ isomers. Two samples have a high E/H (< 4) consistent with a plant rather than aquatic origin (Bondetti et al. 2020).

### GC-C-IRMS

**Fig. 6:**
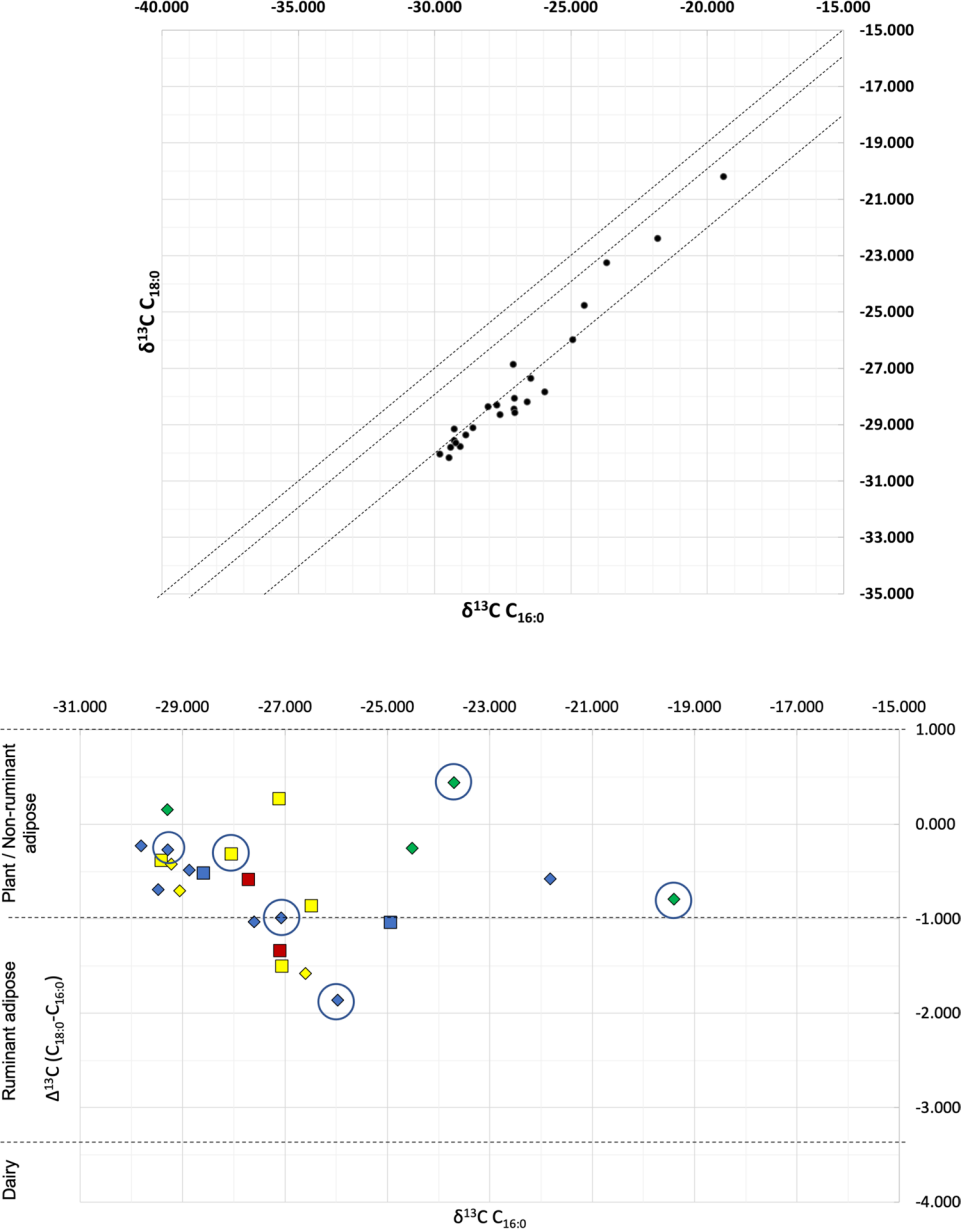
Top — scatter plot of results from the 25 samples analysed by GC-C-IRMS, showing C_16:0_ ẟ^13^C from the C_18:0_ ẟ^13^C. Bottom — plot of Δ^13^C against ẟ^13^C_16:0_ for the 25 ceramic samples from Isla de Mona selected for GC-C-IRMS analysis. Indigenous samples = diamonds. European samples = squares. Lipid profiles represented by colour: Lipid Profile 1 = green, Lipid Profile 2 = yellow, Lipid Profile 3 = blue, Lipid Profile 4 = red. Six samples with SRR/RRR ratio over 75.5%, a possible indication of the presence of aquatic oils, are circled

GC-C-IRMS analyses were carried out on 25 of the 40 sherds included in this study to determine the ẟ^13^C values for the major fatty acids, C_16:0_ (palmitic acid) and C_18:0_ (stearic acid), and assess the potential sources of the lipids extracted through use of the Δ^13^C proxy. The ẟ^13^C values of palmitic and stearic fatty acids reflect their dietary and biosynthetic origin, which allows plant/non-ruminant, ruminant adipose and ruminant dairy fats to be distinguished (Copley et al. 2003; Dunne et al. 2012). The stable carbon isotope data for methyl stearate and methyl palmitate are shown in Fig. 6, and the individual values found for each of the 25 analysed samples were shown in Table 3.

As demonstrated in Fig. 6, most of the Isla de Mona ceramics fall within a relatively tight cluster. Four samples trend towards values associated with either C_4_ plants or aquatic resources (from − 25.0 towards − 15.0); all four of these data points are extracts of Indigenous sherds and are circled in Fig. 6. The range between − 1.00 and 4.00 has been interpreted as indicating plant products or non-ruminant fats (Dunne et al. 2022a, b, p. 267), − 1.00 to − 3.30 indicating ruminant adipose fats, and below − 3.30 indicating ruminant dairy fats (Mukherjee et al. 2008, p. 2068). These values are based on reference samples from a number of regions including South Asia, Europe, and Egypt (Dunne et al. 2012; Dunne et al. 2022a, b; Mukherjee et al. 2008; Steele et al. 2010; Suryanarayan et al. 2021). However, previous investigations using reference animal fats, notably porcine fats, show some overlap in the ẟ^13^C values of lipids for marine organisms and non-ruminant species (Whelton et al. 2021, p. 6), making the interpretation of these samples less clear.

The interpretation of GC-C-IRMS data in vessels that show a clear plant contribution from their lipid profiles is complicated by the fact that fatty acids which derive from plant processing are likely to contribute carbon more depleted in ^13^C and could therefore alter the δ^13^C values of the C_16:0_ and C_18:0_ fatty acids. Plants that utilise the C_4_ photosynthetic pathway (such as maize or millet) are even more depleted in carbon and thus fall further along the range show in Fig. 6 which show the value of C_4_ plants increases to the right of the graph. Most of the Isla de Mona ceramics analysed here show a clear plant contribution in their molecular profiles. Therefore, according to the ‘animal fat proxy’ which discusses how and why plant products may alter isotopic values (Whelton et al. 2021), we must interpret our GC-C-IRMS data with caution as the plant signatures so clearly demonstrated in the GC–MS data are likely to effect the isotopic values found in our GC-C-IRMS analysis. However, given the lipid concentrations found in our samples and the GC–MS data which suggests a plant origin, it could be argued that most of the vessels were used to process plant or, possibly, a mixture of animal fats and plant products.

## Discussion

### Lipid preservation, vessel use, and reuse

A variety of compound classes have been identified in our ceramic samples including saturated and unsaturated fatty acids, dicarboxylic acids, hydroxy fatty acids, *n-*alkanes, terpenoids, phytosterols, and some fatty alcohols. Several of these compound classes, especially long-chain dicarboxylic acids, hydroxy acids, phytosterols, and terpenoids, either originate in plants or represent the degradation products of plant constituents. The similarity amongst these samples tells us two important things: first it indicates that a variety of ceramic forms contained plant-based products at some point, suggesting culinary practices that made use of plant-based products in several different vessel types. Second, the similarities amongst these samples suggest that the presence of these molecules is unlikely to be a result of environmental contamination, given the results of the gradient analysis test performed on four sherds. The relative homogeneity of lipids present could also indicate reuse of these vessels for a variety of foodstuffs throughout the lifespan of these ceramics.

Four ‘lipid profiles’ have been identified, and all apart from Lipid Profile 1 indicate a clear plant contribution. Lipid Profile 1 is not sufficiently diagnostic to provide a secure answer as to what these vessels contained in the past. The fatty acids C_18:0_ and C_16:0_ are ubiquitous across a wide range of foodstuffs. LCFAs are also present in Lipid Profile 1, and while the combination of these compounds in archaeological ceramics has been interpreted as evidence for degraded animals fats (Dudd et al. 1998; Whelton et al. 2018), questions have been raised as to how specific this profile is and whether or not plant oils can be distinguished clearly from animals fats without additional diagnostic compounds present (Steele et al. 2010). Conversely, the VLCFAs present in these samples are more likely to derive from plant waxes than animal fats. Therefore, while there may have been a contribution from degraded animals fats in this lipid profile, there is also strong evidence for a plant contribution.

Lipid Profiles 2, 3, and 4 show abundant evidence for a clear contribution from plant foodstuffs. The high levels of the UFA oleic acid (C_18:1_) found in Lipid Profile 2 could be either be derived from plant oils or could possibly be interpreted as a sign of contamination (Whelton et al. 2021), as oleic acid is likely to rapidly break down in archaeological contexts given the propensity for oxidisation and thermal degradation of this compound (Colombini and Modugno 2009). Similarly, the smaller peaks of erucic acid (C_22:1_) could offer evidence of plants from the *Brassica* family, or oils such as rapeseed oil (Colombini and Modugno 2009), yet concerns that this compound can be present as a laboratory contaminant requires us to interpret it with caution. Some plants from the *Brassica* family are native to Isla de Mona, including *Cakile lanceolata* and *Lepidium virginicum* (Britton 1915), so the identification of erucic acid may indicate the cooking and consumption of these native plants. In addition, the 2-hydroxy fatty acids and long-chain dicarboxylic acids found in Lipid Profile 2 are further evidence for plant products, as 2-hydroxy fatty acids are widely present in plant sphingolipids (Ukawa et al. 2022), while mid- to long-chain dicarboxylic acids are common oxidations product of erucic and oleic acids, both of which are found in high abundance in plants (Colombini and Modugno 2009, p. 8). This strengthens the interpretation of oleic acid and erucic acid as being derived from the past vessel contents rather than contamination. This is also the case for Lipid Profile 3, where odd-numbered *n-*alkanes add to the evidence from the 2-hydroxy fatty acids and long-chain dicarboxylic acids that there is a plant contribution to the molecular profile observed. Long-chain α,ω-dicarboxylic acids (diacids) with a chain length of C_16_ to C_26_ are considered diagnostic for suberin, a compound present in the outer part of underground plants (Dunne et al. 2022a, b, p. 262). Diacids are especially prevalent in Lipid Profiles 2 and 3, further attesting to the use of these ceramics for plant processing and the likely consumption of underground plant organs such as tubers in the local diet of Isla de Mona.

Results from the GC-C-IRMS indicate a possible contribution from a C_4_ plant in two samples of Indigenous pottery (177 and 178). While there is some overlap between C_4_ and marine signals, the lack of marine signals from the GC–MS analysis suggests the processing of a C_4_ plant in these two vessels. Maize is perhaps the best known and most widely studied C_4_ plant grown by Indigenous populations in the Americas, with both native and imported varieties playing an important role in Caribbean diets including in Puerto Rico (Pagán-Jiménez and Mickleburgh 2022). Maize was amongst the many crops gown on Isla de Mona in addition to cassava, beans, peppers, and fruit (Fernandez de Oviedo y Valdés, 1851, bk. XII, chap. VII, p.394; book XVI, chap. I, p. 465; Las Casas 1875, p. XCVIII, 68–69; Wadsworth 1973). Maize was an important agricultural product in the Americas, and the detection of ẟ^13^C values consistent with C_4_ plants found in ceramic vessel can provide further evidence for the consumption and preparation of maize in the Americas (Reber and Evershed 2004). In addition, plants from the *Amaranthus* genus also utilise the C_4_ photosynthetic pathway, and while there are examples of *Amaranthus* native to every continent except Antarctica, the majority are native to the Americas (Waselkov et al. 2018). Wild forms of the *Amaranthus* genus have been identified on archaeological sites in South America that date from between 5910 and 5270 BCE (Arreguez et al. 2013), and recent archaeological evidence confirms that domesticated forms of *Amaranthus* were consumed in New Mexico as far back as the twelfth century CE (Turner et al. 2021). *Amaranthus* is present on Isla de Mona (Britton 1915, p. 40), so it is possible that a species of *Amaranthus* contributed part of the C_4_ plant signal identified in these pots, in addition to the maize cultivated on the island.

The diterpenoids identified in the ceramics in Lipid Profile 4 have an unquestionable plant origin, as these compounds are only synthesised by conifers, most likely from the *Pinaceae* family. Conifers have not been identified in early studies of vegetation on Isla de Mona (Britton 1915), which suggests that these two vessels, both of the ‘olive jar’ ceramic type, were lined with a resin, pitch/tar prior to their arrival on Isla de Mona. While the term ‘olive jar’ appears to indicate a past content of olives or olive oil, studies of Spanish shipping documents have shown that this vessel type was also used to transport wine, water, honey, beans, chickpeas, capers, almonds, dates, pitch, and gun powder (Avery 1997, p. 89). Olive jar 175 contains levels of tartaric acid that indicate this vessel contained wine. This vessel was recovered from Cueva Campanita, where sixteenth-century material culture including a small bronze bell has been recorded and interpreted as evidence for Indigenous Catholic practices (Dávila Dávila 2003, p. 167 and Fig. 16). The archaeological context raises the intriguing possibility that this vessel contained wine used in religious rites. Olive jar 175 is of a shape consistent with the ‘Early’ style of olive jars as defined by Goggin, which were produced between 1500 and 1580 CE (1960; James 1988, p. 44). Given the age of this olive jar, we suggest that the molecular evidence for wine in this vessel would constitute the oldest wine residues discovered in the Americas to date.

### Comparison with faunal remains

Results from GC-C-IRMS indicate a past content that includes plant products/non-ruminant adipose fats, with a minority of samples falling within the range traditional associated with ruminant adipose tissues. There is also a possible contribution from C_4_ plants in at least two samples of Indigenous pottery (177 and 178). The values obtained through this analysis show a relatively tight cluster, with the only clear differentiation being the two Indigenous vessels enriched in C_13_. As there are no large ruminants native to the Greater Antilles, ruminant values in Indigenous pots may indicate that they used these exotic animals in their local cuisine. In addition, faunal remains from sixteenth-century excavated contexts in Isla de Mona include cow, pig, and goat. Mixtures of products, either by intentional mixing or reuse of pottery vessels, complicates the interpretation of GC-C-IRMS results; a mixture of ruminant adipose fats and C_3_ plant products can produce ∆^13^C values similar to non-ruminant fats (Hendy et al. 2018). In some instances, these values have been demonstrated in ceramics where non-ruminant fats were mixed with C_4_ plant products such as maize. Such a mixture is certainly possible for these samples. Furthermore, we would expect not only pig, but also manatee, turtle, and iguana to fall within the ‘plant/non-ruminant adipose’ range yet would be less fatty than pig. The faunal remains of these animals are widely present on Isla de Mona, so it remains a possibility that relatively lipid-poor non-ruminants such as these were processed in some of our samples. However, without reference material to test, it is difficult to establish what ∆^13^C values would be present in the tissues of these animals.

It should also be noted evidence for plant processing in archaeological ceramics is difficult to detect, although several studies have achieved this (Dunne et al. 2021, 2016; Dunne et al. 2022a, b). The reason for this is simple: the lipid concentration of plant oils in edible plant material exists in relatively low proportions when compared to the lipid concentrations found in fat-rich meats (Charters et al. 1997; Evershed 2008). This would cause the signal from animal fats to ‘swamp’ the plant signal in archaeological ceramics in which plant and animal products had been mixed. This makes the possible identification of plant processing noteworthy. Our abundant evidence for plant processing in the Isla de Mona ceramics appears to suggest that plant products were frequently processed within these vessels. 

There is ample evidence that fish were consumed at Isla de Mona based on the numerous fish bones and marine mollusc shells recovered from the site. However, there is only limited evidence for aquatic biomarkers in either the Indigenous vessels or the European vessels. The identification of marine fats is well established in Eurasian pottery assemblages (both using lipid biomarker and compound-specific isotope analysis). Only a handful of our 40 samples (*n* = 6) revealed evidence for aquatic oils through GC–MS, and the isotope data either demonstrates a C_4_ contribution or traces of aquatic oils. Given the limited evidence for aquatic oils from GC–MS data, including the lack of C_20_ APAAs, it appears that these vessels may not have been used to process the fish consumed at the site. Rather fish may have been cooked using a different preparation method. These methods may have included spit roasting, pit roasting, or use of a ‘barbeque’ which is derived from the original Indigenous word ‘barbacoa’ meaning a raised wooden grate on which to smoke food above a fire. Lipids derived from marine-based foodstuffs are often difficult to detect in archaeological pottery as they are high in polyunsaturated fats, and unsaturated fatty acids rarely survive in their unaltered state in archaeological ceramics as they are particularly susceptible to oxidation during burial and during the use of the vessel prior to deposition (Evershed et al. 1992).

The divergence between what residues we have detected in the ceramics and the faunal remains that provide direct evidence for fish consumption are curious, but not without precedent. A recent study conducted on Mesolithic and Neolithic pottery from a site located on the bank of the White Nile in Sudan sought to understand a similar archaeological data set: ample evidence for the consumption of fish was found at the site, yet little to no evidence was found for aquatic biomarkers in the ceramics (Dunne et al. 2022a, b). The authors concluded that fish was likely cooked using a different method, while the ceramics were specialised vessels used to prepare sedges, wild grasses, and leafy plants (Dunne et al. 2022a, b, p. 255). Here, we see a similar phenomenon in that we have detected abundant evidence for plant oils and residues in both European and Indigenous vessels, yet very limited molecular evidence for the many fish which were clearly consumed on this island based on the many fish bones recovered during archaeological excavations.

The GC-C-IRMS data appears to support the results we obtained by GC–MS, in that most of our samples fall within the region of plant/non-ruminant adipose fats found for the Δ^13^C. The interpretation of our GC-C-IRMS data is hindered by a lack of reference material for this region. The recovery of manatee and turtle bones at Isla de Mona suggests that these animals formed part of the diet. Yet, there is currently insufficient data surrounding the isotope values of the lipids found in the tissues of these animals. If we contrast this with the known isotope values for ruminants, non-ruminants, and dairy fats from Europe, the Middle East, and Africa, it becomes apparent how much work is still required to gain a clearer picture of foodways in the early colonial Caribbean.

### Cassava production 

There is abundant evidence of plant products found in the samples from Isla de Mona. The similarities in molecular profiles across a majority of vessel forms raises the possibility of the repurposing of local and imported wares to process plant foods. Cassava (*Manihot esculenta*) is known to have been grown and processed on Isla de Mona, and the intensification of particularly cassava bread production for export in the first half of the sixteenth century may account for the presence of the plant profiles. Cleaning and grating the tubers, removing the juices, grinding the flour, and baking bread are a multi-stage process requiring a range of tools, containers, and cooking wares.

Spanish colonists were incredibly reliant on local Indigenous foodstuffs, which had to be provided by law to labourers and enslaved workers (Alegría, 1990, p. 121). The historically documented intensification of cassava flour and cassava bread production in the first half of the sixteenth century on Isla de Mona may have required an expedient use of all available vessels by Indigenous labourers, including novel imported forms. This marks a shift from traditional subsistence production to a colonial export industry in which a variety of imported and local vessels were given over to this task. So far archaeological investigations have not revealed colonial-era locally produced ceramics in forms specifically related to cassava processing, and this is an avenue for future research. However, until unique biomarkers for cassava can be determined chemically and identified in archaeological ceramics, we can only point to this as a likely source of the plant signals detected in our samples. Future research focus is directed to species specific correlations using detailed reference collection materials gathered on island and more targeted multi-method characterisation techniques.

## Conclusions

The excavations at Isla de Mona yielded ceramic artefacts of both Indigenous and European manufacture from the early colonial period providing us with a unique opportunity to explore the dietary and subsistence practices present on an island at the heart of the Caribbean. This pilot study has shown that ample lipids are preserved in both Indigenous and European ceramics recovered from Isla de Mona. Notably, the divergence between the zooarchaeological evidence from excavations on the island (which suggests an extensive exploitation of marine resources) and the biomolecular evidence from our ceramic samples (which strongly indicates the processing and cooking of plant-based foodstuffs) offers an intriguing insight into the culinary practices on Isla de Mona. Put simply, it appears there was more than one way to cook a fish. While Eurasian pottery from colder climates has yielded ample molecular evidence for the processing and cooking of fish products, our research suggests that at Isla de Mona, fish were prepared in another manner, such as on the ‘barbacoa’. Numerous contemporary sources discuss the use of a ‘barbacoa’ to cook and smoke fish. As a ‘barbacoa’ was a large raised wooden grate, the remains of this cooking apparatus are unlikely to survive in the archaeological record, and the use of a ‘barbacoa’ would help to explain the discrepancy between the faunal evidence for fish consumption and the lack of marine residues in these pots. The formation of APAAs requires not only the presence of fish products in pots but requires that heat be applied to the fish oils as they are prepared in the ceramic vessel. In culinary traditions that favour a hot fish-stew over a barbecued fish fillet, one is far more likely to detect APAAs in ceramic vessels. In a scenario where fish were prepared on a barbecue and perhaps even served in these very ceramic vessels, APAAs would be unlikely to form or be preserved within the ceramic matrix of these vessels as heat would not have been applied to the vessel while it contained fish. This offers an interesting insight into culinary exchange on the island: it appears traditional foodways were maintained even after an influx of European colonialists arrived on the island with their glazed ceramics and olive jars.

The lack of evidence for dairy products in our samples further suggests that European colonialists quickly came to adopt and rely on Indigenous culinary traditions. Even though goat and cow bones have been found in archaeological contexts on the island, we see no evidence for dairy fats in our samples. It seems more likely that these animals were slaughtered for their meat, or if dairying activities did occur on the island, the resulting products were not contained in the selection of pots we have analysed here. While dairy products have long been a staple of European culinary traditions, this does not appear to be the case on Isla de Mona if the 40 ceramics analysed here are representative of local culinary practices. If dairying was absent at Isla de Mona, despite the presence of goats and cows, this may offer further evidence for the continuation of Indigenous culinary traditions in the face of European colonialism and the importation of European ceramic vessels. In the future, a larger study incorporating a greater number of ceramics can help to provide a more comprehensive assessment of this early interaction between Indigenous and European subsistence strategies.

The molecular evidence suggests that rather than relying on European-style fish stews and dairy products, the inhabitants of Isla de Mona largely used their ceramic wares to process plant-based food items. The detection of diacids, hydroxy fatty acids, phytosterols, and diterpenoids are indicative of either C_3_ or C_4_ plants, while two indigenous ceramics seem to have contained plant products derived from an arid-adapted C_4_ plant, such as maize or *Amaranthus*. Every class of ceramic analysed yielded clear evidence for leafy green plant products, except for the two Spanish olive jars included in this study.

The molecular evidence from two Spanish olive jars included in this study suggests that some European culinary traditions persisted. The detection of wine residues in olive jar 175 is significant for two reasons: first, it is the earliest molecular evidence for wine in the Americas yet detected, and second, the discovery of Spanish olive jar 175 inside a cave, near a bell thought to have been used in religious rites, raises the intriguing possibility that imported wine was being consumed on the island. Whether consumed by Europeans or members of the Indigenous population, this is direct evidence for the importation of European wine to a tiny island in the Caribbean shortly after the arrival of Spanish colonialists.

These preliminary interpretations drawn from our small sample set of 40 sherds have demonstrated the potential for a full-scale study of Indigenous and European ceramics from this region. With additional research on a larger assemblage, it will be possible to assess over-arching patterns in diet and culinary practices on Isla de Mona in the period directly following Spanish colonialism in the Caribbean.

## Supplementary information

Below is the link to the electronic supplementary material.

Supplementary file1 (DOCX 147 KB)

## Data Availability

For further information, go to—https://www.springernature.com/gp/authors/research-data-policy/data-availability-statements/12330880 All original scientific data from this research is available to all and can be accessed here: Link to access: https://docs.google.com/spreadsheets/d/1MvVpBVpdrC0ZT5K1mbEfUr1hNC2gvSstYKzZrmul26Q/edit?usp=sharing
