# Supplementary material for: Molecular evidence for new foodways in the early colonial Caribbean: organic residue analysis at Isla de Mona, Puerto Rico
Source: Archaeol Anthropol Sci. 2023 May 3;15(5):70. doi: 10.1007/s12520-023-01771-y (PMC10154268; doi:10.1007/s12520-023-01771-y)
Supplement: Supplementary file 1 — Supplementary file1 (DOCX 147 KB) [file 12520_2023_1771_MOESM1_ESM.docx]

## Supplementary Information 1

TITLE: Molecular evidence for foodways in the early colonial Caribbean: organic residue analysis at Isla de Mona, Puerto Rico

AUTHORS: Lisa Briggs^1,7*^, María Mercedes Martínez Milantchi^2,6^, Oliver E. Craig^3^, Alexandre Lucquin^3^, Jago Cooper^4^, Carl Heron^1^, Alice Samson^5^

AFFILIATIONS:

^1^ Department of Scientific Research, The British Museum, Great Russell Street, London WC1B 3DG, UK

^2^ Department of Africa, Oceania and Americas, The British Museum, Great Russell Street, London WC1B 3DG, UK

^3^ BioArCH, University of York, Environment Building, Wentworth Way, Heslington, York YO10 5NG, UK

^4^ University of East Anglia, Sainsbury Centre, Norfolk Rd, Norwich NR4 7TJ

^5^ University of Leicester, University Road, Leicester, LE1 7RH, United Kingdom

^6*^ Current: Department of Anthropology, Stanford University, 450 Jane Stanford Way, Stanford, CA 94304-2034, USA

^7*^Current: Cranfield Forensic Institute, Cranfield University, College Rd, Cranfield, Wharley End, Bedford MK43 0AL, UK

* Corresponding author: [ebriggs@britishmuseum.org](mailto:ebriggs@britishmuseum.org)

ORCID:

Lisa Briggs 0000-0001-6002-5443

Oliver E. Craig 0000-0002-4296-8402

Alexander Lucquin 0000-0003-4892-6323

Jago Cooper 0000-0002-2396-4414

Carl Heron 0000-0002-5206-7464

In order to remove possible contaminants, the exterior surface of all vessels was removed with a Dremel drill. Where present, sediment (Sample 178) or carbonised food crust (Sample 235) adhering to the vessel surface was removed with a sterilised scalpel and retained in sterilised Au foil. The interior surface was then cleaned with a Dremel drill and a sample of pulverised ceramic powder (2g) was then obtained by drilling into the interior of the vessels to a depth between 2-5 mm of the interior surface (Craig et al., 2005, 2011). All Dremel drill bits and scalpels were cleaned with dichloromethane in between samples.

From each vessel, the 2g of pulverised ceramic powder divided equally and extracted using two extraction methods. First, lipids were extracted and methylated in one step using the acidified methanol method (Correa-Ascencio and Evershed, 2014; Craig et al., 2011) in which 1g of ceramic powder was combined with 4mL of methanol (MeOH) and ultra-sonicated for 15 minutes at 30°C. Next, 80ul of concentrated sulfuric acid (H_2_SO_4_) was added and samples were heated at 70°C for four hours in sealed tubes. After cooling samples were extracted with *n*-hexane (2x) and neutralised with potassium carbonate-packed glass wool. An internal standard was added before extraction (10mg *n*-tetratriacontane) and after extraction (10mg *n*-hexatriacontane).

The remaining 1g of ceramic material was then analysed by a solvent extraction method. In order to assess levels of potential contamination from soil and the deposition environment, as well as the extent to which lipids can penetrate inside the porous ceramic matrix of pottery vessels (Stern et al., 2000), a subset of four sherds were selected for a gradient testing. Each of the four sherds were drilled a depth of 1mm and approximately 0.2g of pulverised ceramic powder was obtained, then this process was repeated an additional four times increasing in depth into the interior of the vessel each time resulting in five sets of ceramic powder from each sherd obtained on a gradient towards the interior of the vessel.

These gradient samples, along with the 1g of ceramic material obtained by drilling 2-5 mm into the ceramic sherds of the other 26 vessels, were extracted by ultrasonication in dichloromethane/methanol (DCM/MeOH, 2:1 v/v, 3 x 5 mL) together with an internal standard (10 µg *n*-tetratriacontane). The resulting total lipid extracts (TLEs) were combined and dried under a gentle stream of nitrogen. Of the 40 ceramic vessels available, a subset of seven were selected for a further extraction by acid butylation in order to assess the presence of tartaric acid. These seven consist of Spanish ‘olive jars’ that may have held wine and Indigenous vessels that may have held liquid contents based on their morphology. This subset was treated for 2 hours at 80°C with a boron trifluoride-butanol/hexane solution (BF_3_-BuOH/hexane, 1:2, v/v). After centrifugation, the solution was collected, neutralised with a saturated solution of sodium carbonate and extracted three times with DCM. The extracts were combined and washed twice with distilled water and evaporated under nitrogen. All the extracts (following solvent extraction and acid butylation) were trimethylsilylated with *N,O*-bis(trimethylsilyl)trifluoroacetamide (BSTFA) with 1% trimethylchlorosilane (70° C, 1h). Ten µg of internal standard (*n*-hexatriacontane) were added to each sample prior to dilution in hexane and immediate analysis by GC-MS.

 Extracts obtained by the acidified methanol method were analysed on an GC-MS instrument (Agilent 5977B coupled with Agilent 7890B) on a HP- 5MS column (30 m × 0.250 mm × 0.25 µm; Agilent Technologies, USA). The oven temperature was set at 50 °C ramping at 10 °C/min to 200 °C then raised at 4 °C/min to 320 °C. TLEs obtained by the solvent extraction and acid butylation method were analysed on an Agilent 5975 coupled with an Agilent 6890 using the same temperature programme as above. The carrier gas was helium. The acidified methanol extracts, TLEs, and acid butylation extracts were analysed at the Scientific Research Department of the British Museum.

The lipid extracts selected for GC-C-IRMS and screening for aquatic oils were analysed at the BioArCH laboratories at the University of York using a GC-MS equipped with a DB-23 (50%-Cyanopropyl)-methylpolysiloxane column (60 m × 0.250 mm × 0.25 µm; J&W Scientific, Folsom, CA, USA). The oven temperature was set at 50 °C for 2 minutes before increasing to 100 °C (10 °C/min). The temperature was then raised by 4 °C/min to 140 °C, then by 0.5 °C/min to 160 °C and, finally, by 20 °C/min to 250 °C where it was maintained for 10 min. The SIM (Selective Ion Monitoring) mode was used in order to target the specific markers of aquatic resources using characteristic ions groups: *m/z* 74, 87, 213, 270 for 4,8,12-trimethyltridecanoic acid (TMTD), *m/z* 74, 88, 101, 312 for pristanic acid, *m/z* 74, 101, 171, 326 for phytanic acid and *m/z* 74, 105, 262, 290, 318, 346 for the detection of *ω*-(*o*-alkylphenyl)alkanoic acids of carbon lengths C_16_ to C_22_ (APAA_16-22_). In addition, separation of the two phytanic acid diastereomers (3S,7R,11R,15-phytanic acid or SRR and 3R,7R,11R,15-phytanic acid or RRR) was obtained which enabled the calculation of the percentage of SRR in total phytanic acid (SRR%) by integrating the *m/z* 101 ion (Lucquin et al., 2016) The carrier gas used was helium with a flow rate of 1.5 mL/min.

Stable carbon isotope values of methyl palmitate (C_16:0_) and methyl stearate (C_18:0_), derived from precursor fatty acids were measured by GC-c-IRMS, following existing procedures from one of our previous papers. An Isoprime 100 (Isoprime, Cheadle, UK) linked to an Agilent 7890B series GC (Agilent Technologies, Santa Clara, CA, USA) with an Isoprime GC5 interface (Isoprime Cheadle, UK) was used, with a DB-5MS ultra-inert fused-silica column (60 m _ 0.25 mm id x 0.25 mm film thickness). Injection was done in splitless mode where the sample was vaporized at 300 ºC. The temperature was set for 0.5 min at 50 ºC, then increased by 25 ºC min^-1^ to 175 ºC, 8 ºC min^-1^ to 325 ºC and held for 20 min. As the carrier gas, ultra-high purity grade helium was used with a flow rate of 3 ml min^-1^. The gas flows eluting from the column was directed through the reactor tube to oxidize all the carbon species to CO_2_. A clear resolution and a baseline separation of the analyzed peaks were achieved. Eluted products were ionized in the mass spectrometer by electron impact and ion intensities of *m/z* 44, 45 and 46 were recorded for automatic computing of the ^13^C/^12^C ratio of each peak in the extracts. Computation was made with IonVantage and IonOS softwares (Isoprime, Cheadle, UK). The results from the analysis are reported in parts per mil (‰) relative to an international standard (V-PDB). Each batch of samples was calibrated using a calibration curve (average R^2^ = 0.998 in 2 batches) based on expected vs. measured δ13C values of n-alkanes and n-alkanoic acid esters international standards (Indiana A7 and F8-3 mixture). More precisely, the accuracy of the instrument was determined on n-alkanoic acid ester standards of known isotopic composition (Indiana standard F8-3, 8 measurements). The mean ± S.D. values of these were -29.92± 0.13‰ and -23.30± 0.10‰ for the methyl ester of C_16:0_ (reported mean value vs. VPDB -29.90 ± 0.03‰) and C_18:0_ (reported mean value vs. VPDB -23.24 ± 0.01‰) respectively.

Each sample was measured in duplicate. Values were also corrected subsequent to analysis to account for the methylation of the carboxyl group that occurs during acid extraction. Corrections were based on comparisons with a standard mixture of C_16:0_ and C_18:0_ fatty acids of known isotopic composition processed in each batch under identical conditions.
